# G protein estrogen receptor as a potential therapeutic target in Raynaud’s phenomenon

**DOI:** 10.3389/fphar.2022.1061374

**Published:** 2022-11-10

**Authors:** Manal Fardoun, Stefania Mondello, Firas Kobeissy, Ali H. Eid

**Affiliations:** ^1^ Department of Pharmacology and Toxicology, Faculty of Medicine, American University of Beirut, Beirut, Lebanon; ^2^ Department of Biomedical and Dental Sciences and Morphofunctional Imaging, University of Messina, Messina, Italy; ^3^ Department of Emergency Medicine, University of Florida, Gainesville, FL, United States; ^4^ Department of Basic Medical Sciences, College of Medicine, QU Health, Qatar University, Doha, Qatar

**Keywords:** raynaud’s phenomenon, estrogen, VSMC, alpha 2C adrenoceptor, GPER, vasoconstriction, gender bias, cardiovascular disease

## Abstract

Exaggerated cold-induced vasoconstriction can precipitate a pathogenesis called Raynaud’s phenomenon (RP). Interestingly, RP is significantly more prevalent in females than age-matched men, highlighting the potential implication of 17β-estradiol (E_2_) in the etio-pathogenesis of this disease. Indeed, we have previously reported that E_2_ stimulates the expression of vascular alpha 2C-adrenoceptors (α_2C_-AR), the sole mediator of cold-induced constriction of cutaneous arterioles. This induced expression occurs through the cyclic adenosine monophosphate → exchange protein activated by cAMP→ Ras-related protein 1→ c-Jun N-terminal kinase→ activator protein-1 (cAMP/Epac/Rap/JNK/AP-1 pathway). On the basis that estrogen-induced rapid cAMP accumulation and JNK activation occurs so rapidly we hypothesized that a non-classic, plasma membrane estrogen receptor was the mediator. We then showed that an impermeable form of E_2_, namely E_2_:BSA, mimics E_2_ effects suggesting a role for the membranous G-protein coupled estrogen receptor (GPER) in E_2_-induced α_2C_-AR expression. Our current working hypothesis and unpublished observations further cement this finding, as G1, a GPER agonist, mimics while G15, a GPER antagonist, abrogates estrogen’s effect on the expression of vascular α_2C_-AR. These, and other observations, highlight the potential of GPER as a tractable target in the management of RP, particularly in pre-menopausal women.

## Introduction

Cold-induced vasoconstriction is a normal physiological reflex reaction taking place at the level of the extremities (Thompson-Torgerson et al., 2007). It is precipitated when noradrenaline binds to and activates α2C adrenergic receptors (α_2C_-AR) on cutaneous arteriolar VSMCs (Chotani et al., 2000; Charkoudian, 2010). This constriction results in blood redirection from the superficial circulation to internal, more vital, body organs. However, when this vasoconstriction becomes exceedingly exaggerated, a condition termed Raynaud’s phenomenon (RP) could ensue (Herrick, 2012). Patients with RP suffer from vasospastic attacks associated with color change, puffiness, and ulcers at the level of the digits (Gerbracht et al., 1985; Heidrich, 2010). More severe cases of RP may cause necrosis and gangrene of the fingers (Saban et al., 1991).

## Evidence linking estrogen to RP

Epidemiological studies show a much higher prevalence of RP in females compared to age-matched males (Maricq et al., 1993; Garner et al., 2015). The ratio of RP-affected premenopausal females to affected age-matched males may reach 9:1 in some studies (Garner et al., 2015; Fardoun et al., 2016). This reflects a gender-based, or biased, factor in RP prevalence (Maricq et al., 1993). Indeed, it has been reported that a female gender is among the risk factors of RP (Garner et al., 2015). Particularly, premenopausal females are much more affected than post-menopausal females (Greenstein et al., 1996). Interestingly, post-menopausal females receiving unopposed estrogen replacement therapy (ERT) are at a higher risk of RP than post-menopausal women not receiving ERT (Mayes, 1999). Furthermore, estrogen has been reported to increase vascular responsiveness (Li et al., 2014), and that vascular responsiveness is higher in young women or female rats of reproductive age as compared to age-matched men or male rats, respectively (Li et al., 2014). Moreover, supplementing male and female rats with estrogen enhanced their vascular responsiveness (Li et al., 2014). Moreover, in premenopausal females, noradrenaline-mediated vasoconstrictor response is elevated during the mid-menstrual cycle (Chan et al., 2001), a phase characterized by higher estrogen level compared to other stages of the cycle. This vascular regulatory role of estrogen, in addition to its thermoregulatory role (Charkoudian and Stachenfeld, 2016), highlight a potential involvement of estrogen in the etio-pathogenesis of RP. These observations, along with other previously discussed observations (Fardoun et al., 2016), suggest a positive association between the female hormone, 17β-estradiol or estrogen (E_2_), and RP (Flavahan, 2008).

## Estrogen receptors in RP

Estrogen exerts its biological effects by activating the classical genomic pathway or the nongenomic rapid signaling pathway (Pedram et al., 2002). The genomic pathway is mediated by the cytoplasmic/nuclear estrogen receptors, ERα and ERβ (Bjornstrom and Sjoberg, 2005; Prossnitz and Maggiolini, 2009). These receptors act as ligand-activated transcription factors and bind to specific response elements in the promoters of target genes, thus regulating their transcription (Bjornstrom and Sjoberg, 2005; Prossnitz and Maggiolini, 2009). On the other hand, the rapid nongenomic effect is mediated *via* the non-classical G-coupled protein estrogen receptor, GPER (Losel and Wehling, 2003; Bjornstrom and Sjoberg, 2005; Prossnitz and Maggiolini, 2009). This rapid estrogenic effect may also induce a cascade of signal transduction pathways that ultimately regulate gene transcription (Bjornstrom and Sjoberg, 2005). Indeed, GPER plays a role in the rapid transcription of several genes (Kanda and Watanabe, 2003; Maggiolini et al., 2004; Hsieh et al., 2007), further implicating GPER in non-canonical estrogen-induced ER-mediated cellular responses.

We previously showed that estrogen potentiates cold-induced vasoconstriction by spatially and functionally rescuing α_2C_-AR (Eid et al., 2007), the sole mediator of cold-induced vasoconstriction (Chotani et al., 2000). This estrogenic effect was attenuated by the pharmacological inhibition of cytoplasmic estrogen receptors (ER), ERα and ERβ. However, bovine serum albumin-conjugated E_2_ (E_2_: BSA), a cell impermeable form of E_2_, was able to induce α_2C_-AR expression (Eid et al., 2007). Furthermore, the stimulation of early downstream players of α_2C_-AR expression signaling pathway in response to estrogen was rapid (Eid et al., 2007; Fardoun et al., 2020). Together, these findings suggest that the membrane GPER mediates, at least partly, estrogen-induced α_2C_-AR expression.

Based on the above, we hypothesized that GPER is the major driver for estrogen’s effect on α_2C_-AR-induced constriction of cutaneous arterioles. Indeed, our unpublished observations further cement this finding, since we found that G1, a GPER agonist, mimics while G15, a GPER antagonist, abrogates estrogen’s effect on the expression of vascular α_2C_-AR. These, and other observations, highlight the potential of GPER as a tractable target in the management of RP, particularly in pre-menopausal women.

## Discussion

It is important to stress that the cellular model we use for our studies is the optimal model. Isolating and culturing primary vascular smooth muscle cells (VSMCs) from human arterioles have always been elusive. However, we succeeded in optimizing the isolation and culture conditions of such a cell line (Fardoun et al., 2020),. These human VSMCs were extracted by non-enzymatic sprouting method from dermal arterioles of a post-circumcision tissue of a newborn boy. Cell purity was verified with flow cytometry using VSMC-specific markers (Fardoun et al., 2020). Only cells between passages 6 and 11 were used in the experiments as the expression and regulation of α_2C_-ARs is similar among these passages. Studies that employ VSMCs isolated from larger arteries or veins cannot be safely used to project clinically or even physiologically relevant conclusions. This is especially important since the vascular bed from which VSMCs are extracted greatly affects their response to estrogen (Dehaini et al., 2018). Thus, estrogen-induced signaling pathways identified in macro VSMCs may not necessarily be valid in micro VSMCs.

A substantial amount of evidence supports the protective role of GPER in the vasculature and in cardiac function. Contextually, GPER-deficient mice show altered cardiac structure and compromised cardiac function (Meoli et al., 2014; Wang et al., 2017), such as enlarged ventricles and impaired systolic and diastolic functions (Delbeck et al., 2011; Wang et al., 2017). Furthermore, GPER activation in hypertensive female mRen2. Lewis rat ameliorated myocardial relaxation and reduced cardiac hypertrophy (Jessup et al., 2010). In vasculature, GPER plays a blood pressure lowering and anti-atherogenic role. Deletion of GPER in female mice resulted in elevated blood pressure and increased atherosclerosis progression (Martensson et al., 2009). Treatment of postmenopausal mice with the synthetic small molecule GPER-selective agonist G-1 attenuated atherosclerosis (Meyer et al., 2015). In addition, intravenous infusion of G-1 resulted in decreased blood pressure of normotensive Sprague–Dawley rats and in acute dilation of preconstricted resistance arteries of the same animal model (Haas et al., 2009). These results suggest a vasodilatory effect of GPER. In fact, genetic linkage studies in humans showed that the GPER gene maps to chromosome 7p22.3. Notably, this region is implicated in arterial hypertension, suggesting a role of GPER in regulating blood pressure (Lafferty et al., 2000).

**FIGURE 1:**
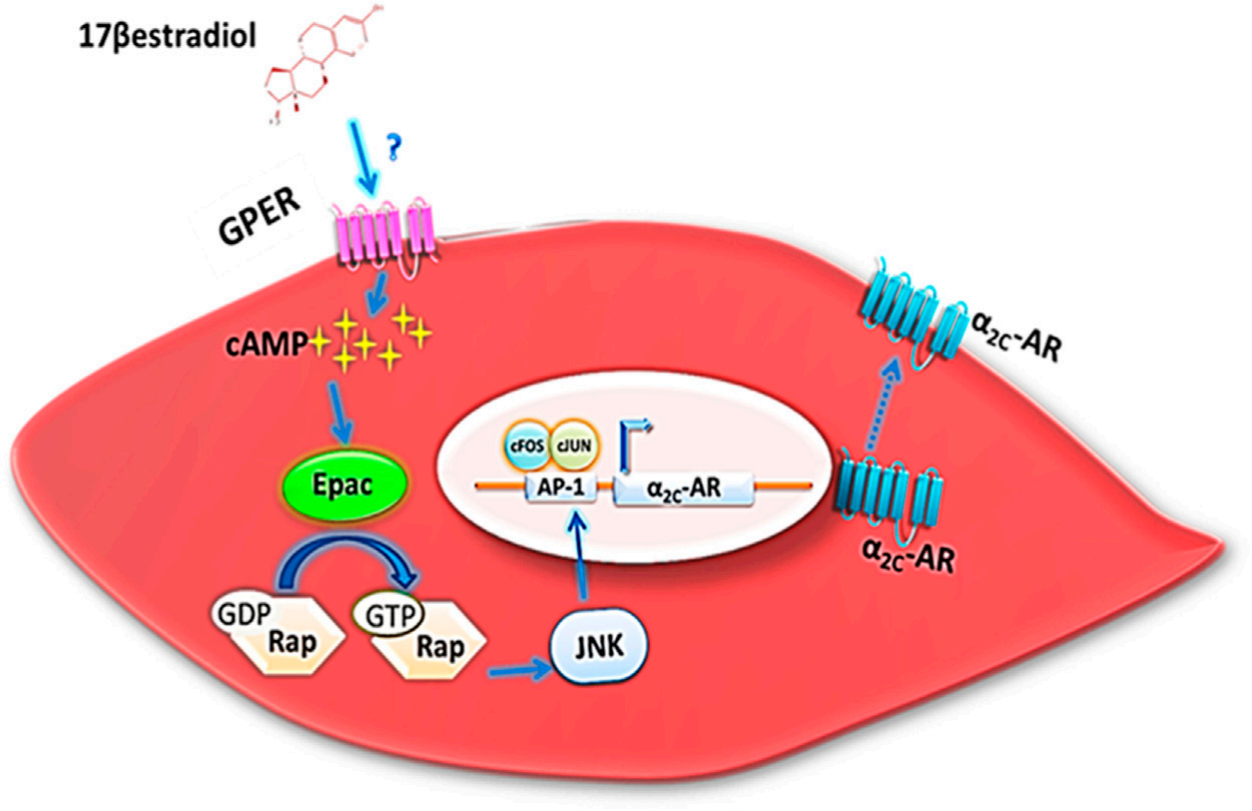
Activation of GPER by estrogen initiates a signaling cascade leading to α_2C_-AR upregulation. GPER mediates estrogen-induced elevation of cAMP level. This elevation is sensed by cAMP downstream effector, Epac. Epac then switches on its target, Rap. Activated GTP-bound Rap induces JNK, which in turn leads to the formation of activator protein (AP-1) by the dimerization of the c-Fos and c-Jun. AP-1 binds to AP-1 site in the α_2C_-AR promoter, initiating its transcription. (α_2C_-AR, alpha 2C-adrenoceptors; cAMP, cyclic adenosine monophosphate; Epac, exchange proteins activated by cAMP; Rap, Ras-related protein 1; JNK, c-Jun N-terminal kinase; AP-1, activator protein-1; GPER, G-protein coupled estrogen receptor; VSMC, vascular smooth muscle cell).

In the context of α_2C_-AR expression and RP, we previously showed that estrogen induced JNK activation within minutes (Fardoun et al., 2020), suggesting that this activation is a rapid non-genomic effect of estrogen. We also demonstrated that estrogen potentiated cold-induced α_2C_-AR translocation *via* JNK activation (Fardoun et al., 2020), suggesting that JNK involvement in this translocation is a result of rapid nongenomic effect of estrogen (Fardoun et al., 2020). Our unpublished observations show that GPER activation induces JNK within the same duration confirming that this estrogen-induced JNK activation is mediated by GPER and thus it is indeed a nongenomic estrogenic response. In addition, this result further confirms that this estrogen-potentiated translocation of α_2C_-AR occurs *via* a GPER-activated JNK-mediated mechanism. Interestingly, activation of GPER by its agonist or by estrogen evokes vasoconstriction in basal renal perfusion pressure (Kurt and Buyukafsar, 2013). However, this vasoconstriction was mediated by a cascade of effectors including p38-mitogen-activated protein kinase (p38-MAPK) and extracellular signal-regulated kinase (ERK1/2) but not JNK (Kurt and Buyukafsar, 2013). It is worth mentioning that GPER mediates estrogen-induced recruitment of the AP-1 to different nucleosomes in promoter of target genes, thus inducing their expression (Li et al., 2010). This becomes more important in light of the fact that we previously showed that estrogen acts through AP-1 to induce expression of vascular α_2C_-AR (Fardoun et al., 2020) (Figure 1). Collectively, these studies introduce GPER as a key player in the signaling pathway mediating RP. Thus, despite the aforementioned cardio- and vasculo-protective roles of GPER, its selective inhibition appears to be a promising therapeutic approach to attenuate RP. Further research is, however, warranted to ensure efficiency and safety of this approach. This is especially important since most of the studies above were either performed *in vitro* (human cells) or in *ex vivo* animal vessels. Owing to the technical and ethical difficulty of isolating and obtaining human arterioles that can be utilized for functional (e.g. myography) studies, the results and hypothesis above will need studies in human arteries before they can be cemented.

## Data Availability

The original contributions presented in the study are included in the article/Supplementary Material, further inquiries can be directed to the corresponding author.

## Abbreviations

AP-1, activator protein-1; α_2C_-AR, alpha 2C-adrenoceptors; cAMP, cyclic adenosine monophosphate; Epac, exchange proteins activated by cAMP; E_2_, 17β estradiol; ERT, estrogen replacement therapy; ERα/β, estrogen receptor α/β; ERK1/2, extracellular signal-regulated kinase; GPER, G-protein coupled estrogen receptor; JNK, c-Jun N-terminal kinase; RP, Raynaud’s phenomenon; Rap, Ras-related protein 1; MAPK, mitogen-activated protein kinase; VSMC, vascular smooth muscle cell.
